# Risk Factors for an Iatrogenic Mallory-Weiss Tear Requiring Bleeding Control during a Screening Upper Endoscopy

**DOI:** 10.1155/2017/5454791

**Published:** 2017-02-27

**Authors:** Shin Na, Ji Yong Ahn, Kee Wook Jung, Jeong Hoon Lee, Do Hoon Kim, Kee Don Choi, Ho June Song, Gin Hyug Lee, Hwoon-Yong Jung, Seungbong Han

**Affiliations:** ^1^Department of Gastroenterology, University of Ulsan College of Medicine, Asan Medical Center, Asan Digestive Disease Research Institute, Seoul, Republic of Korea; ^2^Department of Clinical Epidemiology and Biostatistics, University of Ulsan College of Medicine, Asan Medical Center, Seoul, Republic of Korea

## Abstract

*Background and Aim.* In some cases of iatrogenic Mallory-Weiss tears (MWTs), hemostasis is needed due to severe mucosal tearing with bleeding. Therefore, we aimed to evaluate the risk factors for severe iatrogenic MWTs and the methods of endoscopic bleeding control. *Materials and Methods.* Between January 2008 and December 2012, 426,085 cases of screening upper endoscopy were performed at the Asan Medical Center. We retrospectively analyzed the risk factors for severe iatrogenic MWTs requiring an endoscopic procedure and the treatment modalities of bleeding control. *Results.* Iatrogenic MWTs occurred in 546 cases (0.13%) of screening upper endoscopy in 539 patients. Bleeding control due to severe bleeding was applied in 71 cases (13.0%), and rebleeding after initial bleeding control occurred in 1 case. Multivariate analysis showed that old age, a history of distal gastrectomy, and a less-experienced endoscopist (fewer than 2,237.5 endoscopic procedures at the time of the MWT) were associated with severe iatrogenic MWTs requiring an endoscopic procedure. Among 71 cases requiring bleeding control, a hemoclip was used in 81.7% (58 cases). *Conclusions.* Screening endoscopy procedures should be carefully performed when patients are in their old age and have a history of distal gastrectomy, particularly if the endoscopist is less experienced.

## 1. Introduction

The Mallory-Weiss tear (MWT), first described in 1929, is defined by upper gastrointestinal bleeding from vomiting-induced mucosal lacerations at the esophagogastric junction [1]. In recent series, MWT was found to be the etiology of upper gastrointestinal bleeding in 3% to 10% of cases, even in cirrhotic patients [2, 3]. Usually, the hemorrhaging in MWT is mild, stops spontaneously, and responds to conservative medical management. However, some patients, especially those with stigmata of active bleeding [4, 5], unstable vital signs at admission, and/or associated comorbid disease [6], may require a hemostasis procedure, which is currently best achieved by interventional endoscopy [7].

Iatrogenic complications during gastrointestinal tract endoscopy have become a problem due to recent endoscopic advances and increased endoscopy use. MWT has been recognized as a complication during upper gastrointestinal endoscopy since the first iatrogenic MWT was reported by Watts in 1976 [8], with a recent reported incidence of 0.07%–0.49% [9–11].

Several therapeutic procedures have been applied for the treatment of bleeding MWTs. Many reports showed a higher rate of successful endoscopic hemostasis with hemoclipping, band ligation, and injection therapy [12–16]. In addition, the rates of complications and rebleeding are low after endoscopic treatment of MWTs [5, 12–16]. Bleeding control by an endoscopic procedure has also shown a high success rate and low complication rate for iatrogenic MWTs from upper endoscopy [9, 11]. However, studies into iatrogenic MWTs to date have contained too few cases, and there are not enough reports into the endoscopic treatment modality in iatrogenic MWTs.

To our knowledge, no large-scale study to date has focused on iatrogenic MWTs, particularly in cases requiring bleeding control due to severe bleeding. We thus analyzed cases of iatrogenic MWTs that occurred during endoscopic examination without any preprocedural history of MWT to determine the risk factors for severe bleeding from iatrogenic MWTs requiring endoscopic hemostasis.

## 2. Patients and Methods

### 2.1. Patients

**Figure 1:**
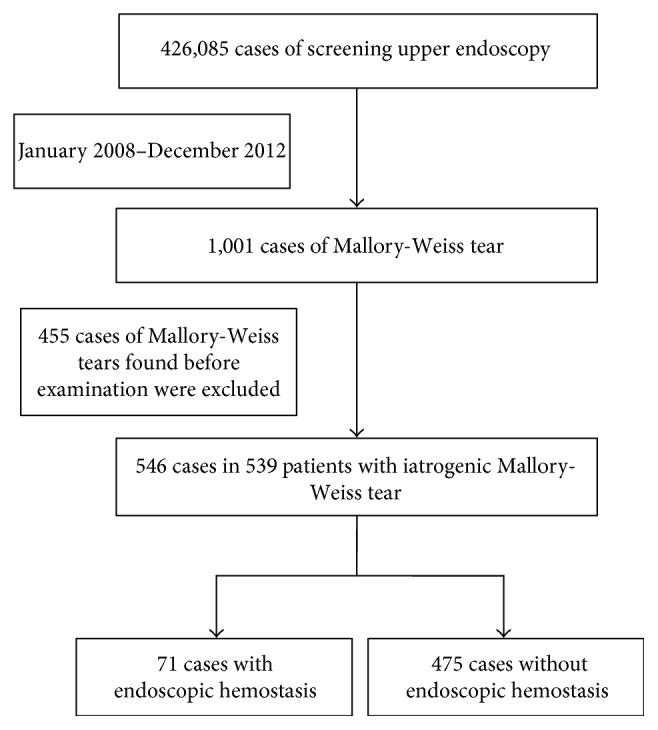
Flow chart of the selection of patients with iatrogenic Mallory-Weiss tears during upper endoscopy.

Between January 2008 and December 2012, 426,085 cases of upper endoscopy screening were performed at the Asan Medical Center; 1,001 patients (659 men, 342 women; median age 54 years, interquartile range [IQR] 43–64 years) were diagnosed with MWTs from this upper endoscopy procedure. From this pool, 455 cases with general MWTs that were found at the start of the endoscopy were excluded. Eventually, 546 cases in 539 patients (327 men, 212 women; median age 52 years, IQR 41.5–65.5 years) with iatrogenic MWTs were enrolled for our present analysis (Figure 1). After reviewing the medical records, we retrospectively analyzed the risk factors for severe iatrogenic MWTs requiring an endoscopic procedure and the treatment modalities for bleeding control. This study was approved by the Institutional Review Board of Asan Medical Center.

### 2.2. Endoscopic Findings and Definitions

**Figure 2:**
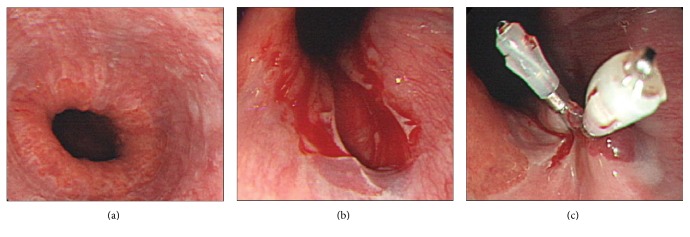
An iatrogenic Mallory-Weiss tear was shown during endoscopic examination and hemostasis using a hemoclip. (a) There were no mucosal tears in the gastrointestinal junction in the initial endoscopy. (b) A mucosal tear occurred during the endoscopic procedure. (c) Two hemoclips were used for bleeding control and to close the torn mucosa.

All endoscopic findings were reviewed by two experienced gastrointestinal endoscopists (H-W.J. and J.Y.A.). Iatrogenic MWT was defined when a tear was not shown in the initial endoscopic findings but occurred during the endoscopic procedure (Figures 2(a) and 2(b)). Bleeding control was applied when the endoscopist decided that severe bleeding or tearing had occurred (Figure 2(c)). General characteristics, including reflux esophagitis, were evaluated. Endoscopy was used to evaluate esophagitis, such as mucosal breakage, which was graded according to the Los Angeles classification [17]. Grade A is one or more mucosal breaks confined to the mucosal folds, each no longer than 5 mm. Grade B is at least one mucosal break more than 5 mm long confined to the mucosal folds but not continuous between the top of two mucosal folds. Grade C is at least one mucosal break continuous between the top of two or more mucosal folds but which is not circumferential. Grade D is a circumferential mucosal break. Rebleeding was defined as fresh hematemesis and/or melena accompanied by symptoms or a fall in hemoglobin levels more than 2.0 g/dL.

### 2.3. Endoscopic Treatment

The endoscopic procedure was performed by endoscopists, with or without conscious sedation. In patients under conscious sedation, midazolam (0.05 mg/kg) was intravenously administered and their cardiorespiratory functions were continually monitored throughout the procedure. Following the administration of local pharyngeal anaesthesia, a single-channel endoscope (GIF-H260; Olympus Optical Co. Ltd., Tokyo, Japan) was used. Bleeding control with metal hemoclips (HX-600-090L or HX-110LR; Olympus Optical Co. Ltd., Tokyo, Japan), 1 : 10000 solution of epinephrine injection, and/or fibrin glue (Beriplast; Aventis Behring Ltd., Marburg, Germany) was performed according to the endoscopist's decision and bleeding severity.

### 2.4. Statistical Analysis

The study results of continuous and categorical variables are presented as median (IQR), with the IQR presented as 25th percentile–75th percentile, and number (percentage), respectively. Categorical variables were compared with Fisher's exact test or Pearson's chi-square test, and continuous variables were compared with a Mann-Whitney *U* test or Student's *t*-test, as appropriate. By using more than one predictor with a significant difference in univariate analysis or with selected variables, multivariate logistic regression analysis was performed to examine the risk factors for poor bleeding control in iatrogenic MWTs. To evaluate the number of previous upper endoscopies performed for bleeding control in iatrogenic MWT cases, the area under the curve (AUC) was calculated using the curve of the number of previous upper endoscopies performed for bleeding control in iatrogenic MWTs. All *p* values were two-sided, and *p* values less than 0.05 were considered statistically significant. All statistical analyses were performed with the SPSS for Windows statistical software package (version 20.0; SPSS, Chicago, IL) and R software version 2.10.1 (https://www.r-project.org).

## 3. Results

Of the 426,085 cases of screening upper endoscopy in our hospital, iatrogenic MWTs occurred in 546 instances (0.13%) in 539 patients. Among these 546 cases, control of severe bleeding was undertaken in 71 cases (13.0%; Figure 1).

### 3.1. Baseline Clinical Characteristics

The clinical characteristics of patients with iatrogenic MWTs are shown in Table 1. Of the total 546 cases, 330 (60.4%) were male and the median age was 53 years (IQR 43.0–64.0). In seven patients, iatrogenic MWTs occurred twice during a screening endoscopy that was performed annually. Distal gastrectomy was performed in 19 cases due to gastric cancer and ulcer and showed more severe iatrogenic MWTs than nonsevere iatrogenic MWTs (11.3% versus 2.3%, *p* < 0.001).

### 3.2. Endoscopic Findings of Patients with Iatrogenic MWTs

**Figure 3:**
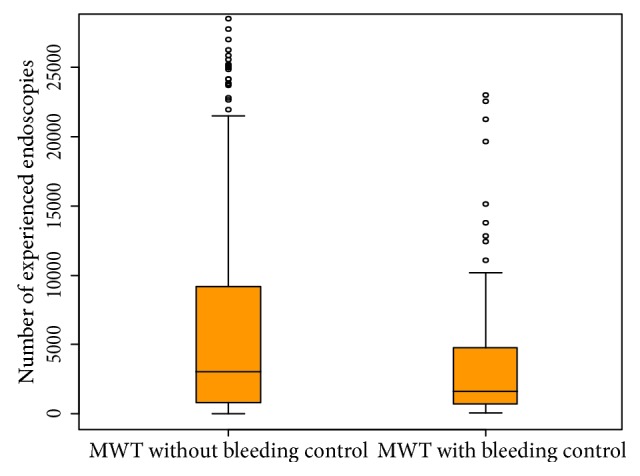
Box and whisker plot of the numbers of previous upper endoscopies performed for bleeding control in iatrogenic Mallory-Weiss tear cases. The upper and lower parts of the whisker represent the observed maximum and minimum, respectively. The upper and lower edges of the boxes represent the 75th and 25th percentiles, respectively. The line inside the box represents the median value. Distributions of the numbers of previous upper endoscopies performed according to bleeding control in iatrogenic Mallory-Weiss tear cases. *MWT*, Mallory-Weiss tear.

**Figure 4:**
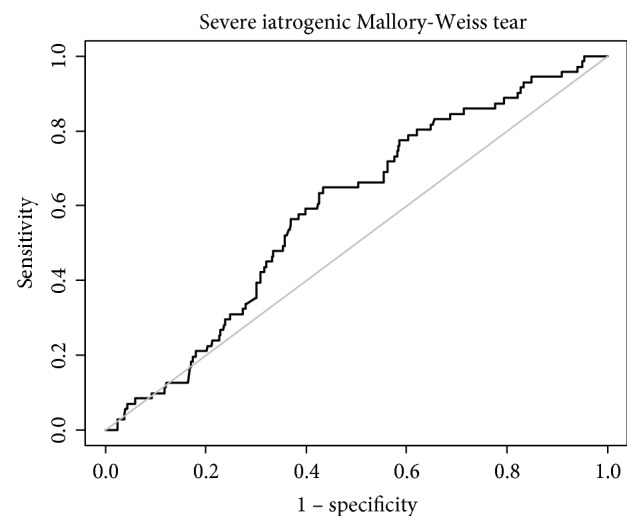
Curve for the number of previous upper endoscopies performed for bleeding control in iatrogenic Mallory-Weiss tear cases. The cut-off for bleeding control is 2,237.5 cases and the area under the curve is 0.590 (95% confidence interval, 0.524–0.655).

Atrophic changes were more frequently found by endoscopy in severe iatrogenic MWTs (74.6% versus 37.9%, *p* < 0.001), and severe iatrogenic MWTs were more frequent for nonsedative endoscopy without midazolam (54.8% versus 36.2%, *p* = 0.004). Severe iatrogenic MWTs were more frequently found for relatively less-experienced endoscopists (a median number of endoscopic procedures performed before the iatrogenic MWT of 1,622 [IQR 699–4,823]) than for experienced endoscopists (a median number of endoscopic procedures before the iatrogenic MWT of 3,037 [IQR 820–9,435]) (*p* = 0.015) (Table 1; Figure 3). Regarding the number of previous upper endoscopies performed for bleeding control of iatrogenic MWTs, the cut-off for bleeding control was 2,237.5 cases and the AUC was 0.590 (95% confidence interval [CI] 0.524–0.655; Figure 4).

### 3.3. Modality of Endoscopic Treatment in Patients with Iatrogenic MWTs

In severe iatrogenic MWTs, a hemoclip was attempted in 58 cases (81.7%) and a median of three clips (IQR 2–4) was used (Table 2). Epinephrine injection alone was tried in nine cases, and a median of 3 cc (IQR 3–5 cc) was used (Table 2). In three cases, a hemoclip (median 2, IQR 1–3) with epinephrine (median 2 cc, IQR 2–4 cc) was used and three hemoclips with fibrin glue (1 cc) were used in one case (Table 2). Rebleeding after initial bleeding control occurred in one patient (0.18%), a 44-year-old male with a history of liver cirrhosis due to hepatitis B. A screening endoscopy was performed for the initial liver transplant work-up, and prothrombin time prolongation was found. At the initial endoscopic procedure, two hemoclips were used for bleeding control and rebleeding occurred after 4 days and was treated using three additional hemoclips.

### 3.4. Risk Factors for Poor Bleeding Control in Patients with Iatrogenic MWTs

Univariate analysis showed that age, a history of distal gastrectomy, a lack of sedation during endoscopy, and a less-experienced endoscopist (fewer than 2,237.5 endoscopic procedures at the time of the MWT) were significantly associated with severe iatrogenic MWTs at the 10% significance level (Table 3). Multivariate analysis with backward elimination showed that the odds ratios of severe iatrogenic MWTs were 2.121 (95% CI 2.014–2.229; *p* < 0.001) for age, 3.213 (95% CI 1.194–8.649; *p* = 0.021) for a history of distal gastrectomy, and 2.118 (95% CI 1.239–3.622; *p* = 0.006) for a less-experienced endoscopist (fewer than 2237.5 endoscopic procedures at the time of the MWT) (Table 3).

## 4. Discussion

Iatrogenic complications during gastrointestinal tract endoscopy have become a problem due to recent endoscopic advances and increased endoscopy use. Since the first iatrogenic MWT was reported by Watts in 1976 [8], the MWT has been recognized as a complication during upper gastrointestinal endoscopy, with a reported incidence of 0.07%–0.49% [9–11]. Our present study findings showed that iatrogenic MWTs occurred in 0.13% of screening upper gastrointestinal endoscopies and bleeding control was needed in 13.0% of these cases. Old age, history of distal gastrectomy, and fewer than 2,237.5 endoscopic procedures at the time of the MWT were the risk factors for severe iatrogenic MWTs requiring an endoscopic procedure. Thus, we should perform screening endoscopy carefully when patients are in their old age and have a history of distal gastrectomy, particularly when the endoscopist is less experienced.

A previous report showed that the characteristics and management of bleeding from iatrogenic MWTs do not differ from those of other etiology-induced MWTs [11]. However, there have been no data on the risk factors for severe iatrogenic MWTs requiring endoscopic hemostasis until now. In our present results, we first showed using multivariate analysis that old age and distal gastrectomized state were risk factors for severe iatrogenic MWTs. In our patients with distal gastrectomy, the volume of the remnant stomach was small and the linear anastomosis resulted in a narrower upper third of the stomach. Hence, tension due to inflation can be increased compared with the normal stomach, and the chance of a severe MWT is high. In addition, our results further showed that the number of endoscopic procedures performed by the endoscopist was one of the risk factors for severe iatrogenic MWTs, with a cut-off of 2,237.5 cases. Even though an iatrogenic MWT could be due to a patient's noncooperation, endoscopies should be carefully performed, particularly when the endoscopist is less experienced.

In most cases, bleeding from an MWT is not severe and stops without an intervention. However, some studies have reported severe bleeding from an MWT requiring hemostasis and hospitalization [4–7]. In iatrogenic MWTs, most cases had a benign clinical course without the need for endoscopic hemostasis and/or blood transfusion [9] and the treatment outcome was not different from those of other causes of MWTs [11]. The risk factors for rebleeding in MWTs have been reported to be bleeding tendency and/or low hemoglobin level [4, 6]. However, the prognosis of bleeding from MWTs caused by endoscopic examination has not been clearly demonstrated. Our present study showed that only one patient (0.18%) had rebleeding from an iatrogenic MWT after endoscopic hemostasis. This patient had a bleeding tendency with a prolonged prothrombin time from liver cirrhosis. Therefore, the risk of further problems in iatrogenic MWTs and rebleeding in severe cases was extremely low. These findings can help with the proper management of patients with MWTs during screening endoscopy.

Bleeding from MWTs can typically be managed by endoscopic hemostasis, and various methods, such as hemoclipping [5, 14–16], band ligation [12, 13, 18, 19], injection therapy [5, 12, 14], electrocoagulation [19, 20], and selective arterial embolization [21, 22], produce high rates of bleeding control without complications. One report that used hemoclips to treat iatrogenic MWTs showed a 100% bleeding control rate without rebleeding or complications [11]. In our current data, we used a hemoclip alone in 81.7% of cases with bleeding, with a median number of three clips. Besides a hemoclip, an epinephrine injection alone, a hemoclip with epinephrine, and a hemoclip with fibrin glue have been applied for bleeding control in iatrogenic MWTs. As shown in this study and previous studies, a hemoclip with or without injection therapy could be considered as a first-line method of treatment for an MWT when tearing and bleeding is severe.

Our current study has some noteworthy limitations associated with its retrospective design. Moreover, we did not assess patients who did not have an MWT during a screening endoscopy when determining the risk factors for iatrogenic MWTs. Nevertheless, our findings provide an impetus for future prospective randomised studies with proper methodological designs for determining the risk factors for severe iatrogenic MWTs.

## 5. Conclusion

Iatrogenic MWTs occur in about 0.13% of screening upper endoscopies and severe bleeding occurs in 13.0% of iatrogenic MWTs. However, endoscopic management of severe MWTs can be successfully performed without complications. Endoscopic examinations should be carefully performed to prevent severe iatrogenic MWTs when patients are in their old age and have had a distal gastrectomy, particularly when less-experienced endoscopists are performing the procedure.

## Figures and Tables

**Table 1:** Baseline characteristics and endoscopic findings of patients with iatrogenic Mallory-Weiss tears.

	Total (*n* = 546)	MWTs with bleeding control (*n* = 71)	MWTs without bleeding control (*n* = 475)	*p* value
Median age, years (IQR, years)	53 (43–64)	63 (53–71)	51 (42–62)	<0.001
Sex (M/F)	330/216	49/22	281/194	0.120
Diabetes mellitus	43 (7.9)	6 (8.5)	37 (7.8)	0.814
Hypertension	130 (23.8)	22 (30.9)	108 (22.7)	0.136
Liver disease	38 (6.9)	4 (5.6)	34 (47.9)	0.805
Medication				
Antiplatelet	35 (6.4)	5 (7.0)	30 (6.3)	0.795
Anticoagulant	6 (1.1)	2 (2.8)	4 (0.8)	0.177
History of distal gastrectomy	19 (3.5)	8 (11.3)	11 (2.3)	0.001
Reflux esophagitis				0.991
None/minimal	397 (72.7)	50 (70.4)	347 (73.1)	
LA-A	109 (19.9)	15 (21.1)	94 (19.8)	
LA-B	33 (6.0)	5 (7.1)	28 (5.9)	
LA-C	7 (1.3)	1 (1.4)	6 (1.2)	
Sedation (yes/no)	335/211	32/39	303/172	0.004
Median endoscopic procedures performed at the time of the MWT (IQR)	2,596 (778.5–8,742)	1,622 (699–4,823)	3,037 (820–9,435)	0.015

*n*: number; MWT: Mallory-Weiss tear; IQR: interquartile range; M: male; F: female; LA: Los Angeles classification. Data represent the numbers of patients (%).

**Table 2:** Modality of endoscopic treatments in patients with iatrogenic Mallory-Weiss tears.

Modality	MWTs with bleeding control (*n* = 71)
Hemoclip	58 (81.7)
Epinephrine injection	9 (12.7)
Hemoclip with epinephrine injection	3 (4.2)
Hemoclip with fibrin glue injection	1 (1.4)

MWT: Mallory-Weiss tear; *n*: number. Data represent the numbers of patients (%).

**Table 3:** Univariate and multivariate analysis of the risk factors for bleeding control in patients with iatrogenic Mallory-Weiss tears.

	Univariate analysis		Multivariate analysis	
	Odds ratio (95% CI)	*p* value	Odds ratio (95% CI)	*p* value
Age	1.005 (1.003–1.007)	<0.001	2.121 (2.014–2.229)	<0.001
Sex (male)	1.048 (0.989–1.109)	0.114		
Diabetes mellitus	1.010 (0.909–1.122)	0.847		
Hypertension	1.053 (0.985–1.125)	0.128		
Liver disease	0.974 (0.871–1.088)	0.639		
Antiplatelet	1.014 (0.903–1.138)	0.816		
Anticoagulant	1.228 (0.937–1.609)	0.137		
History of distal gastrectomy	1.352 (1.161–1.574)	<0.001	3.213 (1.194–8.649)	0.021
Reflux esophagitis	1.015 (0.953–1.082)	0.643		
Sedation	0.915 (0.863–0.969)	0.003	1.233 (0.708–2.147)	0.460
Fewer than 2,237.5 endoscopic procedures at the time of the MWT	1.102 (1.041–1.154)	0.001	2.118 (1.239–3.622)	0.006

CI: confidence interval; MWT: Mallory-Weiss tear.
